# The pH and pCO_2_ dependence of sulfate reduction in shallow-sea hydrothermal CO_2_ – venting sediments (Milos Island, Greece)

**DOI:** 10.3389/fmicb.2013.00111

**Published:** 2013-05-08

**Authors:** Elisa Bayraktarov, Roy E. Price, Timothy G. Ferdelman, Kai Finster

**Affiliations:** ^1^Department of Biogeochemistry, Max Planck Institute for Marine MicrobiologyBremen, Germany; ^2^Center for Marine Environmental SciencesBremen, Germany; ^3^Department of Biosciences, Section for Microbiology, University of AarhusAarhus C, Denmark; ^4^Department of Physics and Astronomy, Stellar Astrophysics Centre, University of AarhusAarhus C, Denmark

**Keywords:** sulfate reduction, sulfate reduction rate, shallow-seahydrothermal vents, pH effect, pCO_2_ effect, microbial activity, extreme environment

## Abstract

Microbial sulfate reduction (SR) is a dominant process of organic matter mineralization in sulfate-rich anoxic environments at neutral pH. Recent studies have demonstrated SR in low pH environments, but investigations on the microbial activity at variable pH and CO_2_ partial pressure are still lacking. In this study, the effect of pH and pCO_2_ on microbial activity was investigated by incubation experiments with radioactive ^35^S targeting SR in sediments from the shallow-sea hydrothermal vent system of Milos, Greece, where pH is naturally decreased by CO_2_ release. Sediments differed in their physicochemical characteristics with distance from the main site of fluid discharge. Adjacent to the vent site (*T* ~40–75°C, pH ~5), maximal sulfate reduction rates (SRR) were observed between pH 5 and 6. SR in hydrothermally influenced sediments decreased at neutral pH. Sediments unaffected by hydrothermal venting (*T* ~26°C, pH ~8) expressed the highest SRR between pH 6 and 7. Further experiments investigating the effect of pCO_2_ on SR revealed a steep decrease in activity when the partial pressure increased from 2 to 3 bar. Findings suggest that sulfate reducing microbial communities associated with hydrothermal vent system are adapted to low pH and high CO_2_, while communities at control sites required a higher pH for optimal activity.

## INTRODUCTION

Microbial sulfate reduction (SR) is a dominant process for the anaerobic degradation of organic material in marine sediments where organic carbon or hydrogen gas serve as the electron donors and are oxidized to CO_2_ and water, while sulfate is reduced to hydrogen sulfide (Jørgensen, 1982). SR also plays an important role in deep-sea hydrothermal vent microbial processes, and has a significant impact on sulfur biogeochemistry at high temperatures (Jørgensen et al., 1992). The influence of temperature on SR is well documented (Isaksen et al., 1994; Knoblauch and Jørgensen, 1999; Finke and Jørgensen, 2008; Hubert et al., 2009). However, our understanding of the effects of pH and CO_2_, two important parameters that significantly impact microbial community structure and function is limited.

Submarine hydrothermal vents are well known for the extreme geochemical conditions that they impose on their inhabiting microbiota. Important selecting factors are low pH, high temperatures and high metal and sulfide concentrations in the hydrothermal fluids (Jannasch and Mottl, 1985; Frank et al., 2013). Furthermore, steep temperature gradients demand adaptations to variable temperature regimes. Cultivation-based studies have shown that sulfur compounds are important substrates for microbes in hydrothermal vent systems (Jannasch and Mottl, 1985).

Most studies of sulfate reducing bacteria (SRB) have focused on environments at circumneutral pH including marine and freshwater sediments (Widdel, 1988; Hao et al., 1996). However, there is now increasing evidence for SR to occur also in low pH habitats, such as acidic lakes and rivers, acidic soils, peat lands, acid rock, or mine tailings (Koschorreck, 2008 and references therein). SR was also observed in hydrothermal systems where the pH is lowered due to CO_2_ venting (Amend et al., 2004) but further knowledge on the activity of these microbial communities is limited. SR has a low yield of metabolic energy (Widdel, 1988). However, surprisingly SRB are active under acidic conditions where additional energy requirements to keep an elevated intracellular pH (Martin, 1990; Lowe et al., 1993), the toxicity of metabolic products (e.g., H_2_S or protonated fatty acids, Hao et al., 1996) and competition with other more resistant microbes (e.g., iron reducing bacteria or methanogens) may inhibit SR to occur (Koschorreck, 2008). Below a pH of 5, the metabolic product of SR is exclusively present in its undissociated form H_2_S which is considered as the most toxic form of sulfide (Moosa and Harrison, 2006). As uncharged gas, H_2_S permeates the cell membrane where it can react with free metal ions and metal containing functional groups of the electron carrier system of the cell (Hao et al., 1996), amino acids and metabolic coenzymes inhibiting the functionality of the microbial cell (Koschorreck, 2008). Volatile fatty acids (VFA) are fermentation products of organic carbon and represent typical substrates of sulfate reducers. VFA exist as protonated organic acids under low pH. In their protonated form they can diffuse through the cytoplasmic membrane and act as “uncouplers” (Bruun et al., 2010) leading to a collapse of the membrane potential (Baronofsky et al., 1984). In addition, protonated organic acids decrease the pH of the cytoplasm upon entering by diffusion. Free intracellular protons can impair processes such as DNA transcription, protein synthesis, and enzyme activities (Baker-Austin and Dopson, 2007). In nature, intermediates of fermentation, e.g., VFA and H_2_, are generally maintained at low concentrations, showing a close coupling of terminal oxidation to fermentation (Finke and Jørgensen, 2008) counteracting an accumulation of acid and a subsequent decrease in pH.

In addition, SR at low pH is of special interest because this process can be applied for biogenic neutralization of acid rock drainage environments and for bioremediation (Kaksonen and Puhakka, 2007). The investigation of CO_2_ venting hydrothermal sediments is of particular interest, as they represent a system in which the degassing-effects of previously sequestered CO_2_ can be investigated (Shitashima et al., 2008). Based on all these implications further knowledge is necessary to understand the biological and chemical dynamics in acidified sediments.

This study was conducted on sediments collected from a shallow submarine hydrothermal vent system in the Aegean Sea near the island of Milos (Greece), and focuses on the effect of pH and pCO_2_ on the activity of SRB. By incubation experiments involving the addition of electron donors and at different pH and pCO_2_, SR activities of hydrothermally influenced sediments were compared to activities in control sediments with higher pH and no CO_2_ venting.

## MATERIALS AND METHODS

### STUDY SITE

**FIGURE 1:**
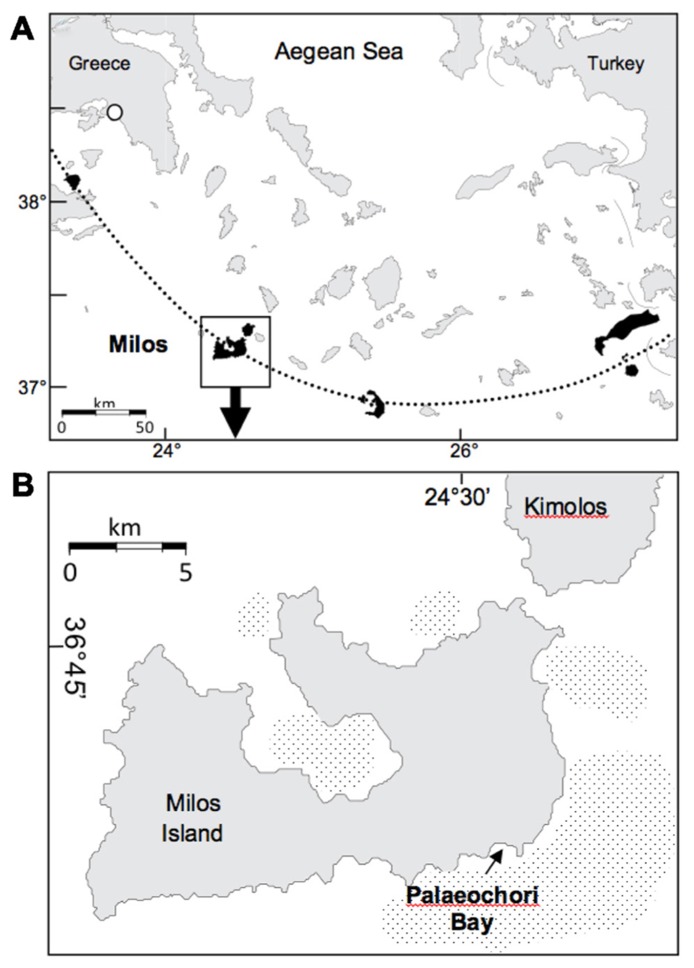
**(A)** Location of Milos Island and other calc-alkaline volcanoes (shaded) along the Aegean Island Arc (dotted line). **(B)** Milos Island and the location of Palaeochori Bay. Stippled offshore areas around the island are mapped gas emissions by echo sounding (Dando et al., 1995). Maps modified from Price et al., 2012.

**FIGURE 2:**
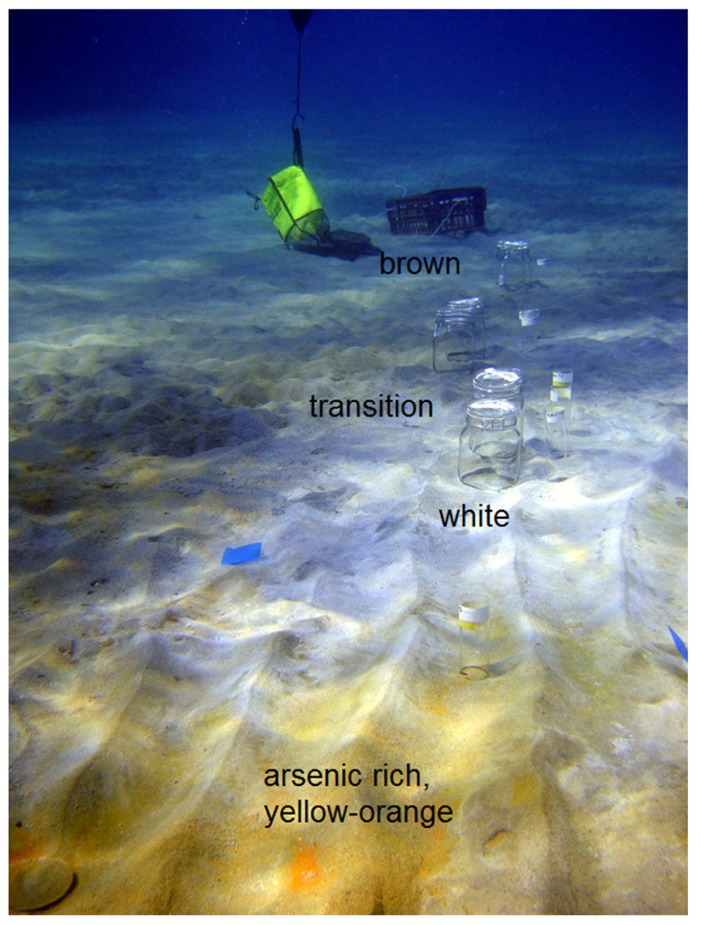
**Transect through the different zones of Paleochori Bay marked with sample bottles**. Zones were divided in yellow-orange, white, transition, and brown. The yellow-orange zone was located adjacent to the venting site.

The study site (36° 40^′^ 25N, 24° 30^′^ 58E; **Figure 1A**) was located ~1 m from an active gaseous hydrothermal vent system at 4.5 m water depth in Paleochori Bay, Milos Island, Greece. The bay is situated at the southeastern coast of Milos in the Aegean Sea (**Figure 1B**). Free gases sampled from the site (collected in 2011) had a mean gas composition of 92.5% CO_2_, 0.13% O_2_, 0.67% N_2_, 7 ppm He, 11450 ppm H_2_, 0.7 ppm CO, and 916 ppm CH_4_ (Price et al., 2013, this issue). These free gas values can be used as an approximation for the discharged gases of the seep. The vent sites displayed a characteristic zonation of colored surface deposits surrounding the gas outlets (**Figure 2**). The sediment next to the vent was covered by a layer of bright yellow-orange deposits containing the arsenic sulfide minerals similar to orpiment (Price et al., 2012). A zone covered by white flocculent material on top of black sediment termed “white zone” surrounded this area. The white precipitate consists of a mixture of amorphous silica and native S (Fitzsimons et al., 1997). It is assumed that this precipitate is associated with microbial mat communities (Dando et al., 1995; Fitzsimons et al., 1997). The white zone was surrounded by an area of gray sediment referred to as the “transition zone” and a brown background zone called the “brown zone” with hydrothermally unaffected sediment characteristics such as ambient temperature and pH. Sediments from the brown zone were used as a control.

### SAMPLING

Sampling was carried out along a transect through different sediment zones by SCUBA diving in August 2010. Sediments at increasing distance (ca. 1, 2, and 5 m) from the gas emission zone were sampled underwater with 1 L preserving jars (**Figure 2**; J. WECK GmbH & Co. KG, Wehr, Germany) containing a butyl rubber stopper which prevented the contamination of the sample with oxygen. Samples were maintained dark and cold during shipping. Temperature measurements were conducted *in situ* at a sediment depth of ~10 cm with a digital thermometer in a custom underwater housing (Fisher Scientific, Germany). The pH measurements integrated over 60 mL of sediment pore water. Measurements were performed ~1 h after sampling from a 10 cm sediment depth at the main sampling locations using 60 mL syringes elongated by tubing connected to long perforated pipette tips. Pore water from the white zone was sampled down to 12 cm sediment depth at 2 cm resolution for analysis of sulfide, sulfate, alkalinity and pH using rhizon soil moisture samplers (5 and 10 cm rhizons, pore size: 0.1 m, Rhizon core solution sampler (CSS), Rhizosphere Research Products, The Netherlands). For sulfide analysis, pore water samples were fixed with an equal volume of 50 mmol L^-^^1^ zinc acetate (ZnAc) solution.

### PHYSICOCHEMICAL MEASUREMENTS

Pore water sulfide fixed with ZnAc was analyzed spectrophotometrically according to Cline (1969). Concentrations of ${SO}_{4}^{2-}$ were measured on a Metrohm Compact 761 ion chromatograph equipped with a Metrohm Metrosep A column. The eluent was a 3.2 mmol L^-^^1^ Na_2_CO_3_/1 mmol L^-^^1^ NaHCO_3_ solution and the flow rate was 0.7 mL min^-^^1^. The standard deviation of repeated measurements was always below 2% of the measured concentration. Prior to measurement, samples were diluted 100-fold with distilled water. Blanks were used for background corrections.

### MEDIUM

Artificial seawater medium for SRB (modified from Widdel, 1988) contained (mmol per liter): KBr (0.756), KCl (8.05); CaCl_2_^⋆^2 H_2_O (10); MgCl_2_^⋆^6 H_2_O (27.89); MgSO_4_^⋆^7 H_2_O (11); NaCl (451); NH_4_Cl (4.67) and KH_2_PO_4_ (1.47) and was prepared without the addition of NaHCO_3_ as buffering solution to allow for easier pH adjustment. For incubation experiments the pH was adjusted to values of 3, 4, 5, 6, and 7 with sterile 1 mmol L^-^^1^ phosphoric acid (H_3_PO_4_) or NaHCO_3_ (Widdel, 1988) before sterilization for 25 min at 121°C and re-adjusted if necessary. Prior to sterilization, needles were inserted into the butyl stoppers of each medium bottle to allow escape of oxygen from the liquid at high temperature. The 75°C hot medium was allowed to cool down under constant stirring and flushing the headspace of the bottle with a mixture of N_2_/CO_2_ at a 9/1 ratio(v/v). The salinity of the SRB-media was 33.

### SULFATE REDUCTION RATE MEASUREMENTS

To determine sulfate reduction rates (SRR) for the different incubation experiments, 20 μL of ^35^S-sulfate tracer containing 100 kBq were injected into 15 mL Hungate tubes containing 2 mL homogenized sediment and 3 mL of SRB medium in 1:1.5 (v/v). Samples were incubated at 40 or 75°C. Per experiment, three killed controls were prepared by transferring the sediment and the medium directly to Hungate tubes containing 5 mL of 20% (w/v) ZnAc solution in centrifuge tubes. The tubes were shaken and tracer was added to the killed slurries. Every sample was prepared in triplicate. Incubations were stopped by transferring the sediment slurries into centrifuge tubes containing 5 mL of a 20% (w/v) ZnAc solution.

Reduced inorganic sulfur compounds were removed from the fixed samples by single step cold acidic chromium distillation method as described by Fossing and Jørgensen (1989) and further modified by Kallmeyer et al. (2004). The ^35^S incorporated into the pool of total reduced inorganic sulfur was recovered as zinc sulfide in traps containing 7 mL of a 5% (w/v) ZnAc solution and finally counted in a scintillation counter (Packard Tri-Carb Liquid Scintillation Counter, MA, USA). SRR were determined as described in Kallmeyer et al. (2004). The porosity of each sediment type was determined by differential weighting of wet and dry sediment after drying for 2 days at 80°C to constant weight.

### INCUBATIONS

All collected sediment samples were used for experiments on residual sulfate reducing activity 52 days after sampling as this was the time required for shipping of sampled material and installation of experimental setup. A pre-incubation for 18 h without tracer was followed by tracer incubation for 35 h. Incubations were performed either at 40 or 75°C. A SRB medium with 11 mmol L^-^^1^ of sulfate and pH 5.3 was used to mimic the *in situ* characteristics of the white zone sediment.

A device for anaerobic slurry preparation and simultaneous pH measurement was constructed of sterilized material (15 min at 121°C). Slurry of 1:4 (v/v) ratio of sediment to medium was prepared and the pH was adjusted with phosphoric acid or NaHCO_3_. After pH adjustment, 5 mL of slurry were transferred into pre-flushed oxygen-free Hungate tubes. The headspace of each tube was flushed with a mixture of N_2_ and CO_2_ at a 9/1 ratio (v/v) and the tube was sealed with a butyl rubber stopper and kept in place by a screw cap.

For experiments on stimulated SR, a mix of VFA (Widdel, 1988) was supplied to the sediment samples as additional electron donors. It contained formate, acetate, propionate, butyrate and succinate in equal-molar amounts providing a total fatty acid concentration of 1 mol L^-^^1^. The mixture of VFA was added with a N_2_/CO_2_-flushed 1 mL syringe through the butyl stopper of the slurry bottles to produce a final slurry concentration of 1 mmol L^-^^1^ VFA. The pH was re-adjusted for each slurry bottle after fatty acid addition. For each experiment with VFA-supplemented slurries, three incubations were prepared as killed controls. All experiments were carried out in triplicates.

For pH experiments, samples were incubated for 16–18 h at 40°C after a short pre-incubation. Pre-incubation time was calculated as the time required for pH adjustment and the distribution of slurry into Hungate tubes prior to incubation with radioactive tracer. Sample preparation was conducted at room temperature and was completed in less than 4 h. For all incubation experiments, the surface sediment fractions (0–5 cm) of white and transition zone sediments and a deeper fraction (5–10 cm) of the brown zone sediment were used. For incubation experiments at pH of 3, 4, 5, 6, and 7, the phosphate buffering system, with pK*a* values 2.15, 6.87, and 12.33 (Perrin, 1972) was appropriate. Before and after each incubation experiment, the pH of each tube was measured after gentle mixing. For pH measurement prior to incubation, 200 μL of the liquid were removed with a needle and a N_2_/CO_2_-flushed 1 mL syringe through the butyl stopper of the tube. The pH was measured after the incubation by inserting the pH electrode into each tube after gentle shaking. All pH values were determined in triplicates. The pH changed during the incubation between ± 0.1 and ± 0.5 pH units (horizontal error bars in **Figure 4**).

Additionally, incubation experiments were conducted to investigate the combined effects of pH and pCO_2_ on SR. Therefore, different CO_2_ partial pressures in the range of 0, 1, 2, and 3 bar were applied after a pre-incubation for 12 h at 40°C. Prior to incubation experiments, CO_2_ partial pressures were adjusted to the gas phase of tubes (10 mL headspace in a total volume of 15 mL) using a pressure reducer.

### STATISTICAL ANALYSES

Arithmetic means and standard errors were calculated for each triplicate set of experiments and are represented by vertical error bars in the activity diagrams (**Figures 4** and **5**). Statistical analysis was performed with SigmaPlot, 12.0, Copyright © 2011 Systat Software, Inc. A One-Way-ANOVA was used to test for statistically significant differences between the groups considering a triplicate at a certain pH value (3, 4, 5, 6, and 7) or CO_2_ partial pressure (0, 1, 2, and 3 bar) as a group. The dependent variable was SRR. Only values above the reported detection limit of 0.04 nmol ${SO}_{4}^{2-}$ cm^-^^3^ d^-^^1^ were taken into account and values below this limit were set to 0. Degree of freedom df, F factor, and *P*-value (df, F, and P) are given. A Two-Way ANOVA was applied to test for differences between groups and treatment (untreated or supplemented sediment).

## RESULTS

### GEOCHEMICAL CHARACTERIZATION

*In situ* measurements of temperature and *ex situ* determination of pH demonstrated that the shallow-sea hydrothermal vent site constituted an extreme environment. The pH decreased with increasing proximity to the gas vent reaching values of 5.3 in the white and 7.6 in the pore water of brown zone sediment (zonation displayed in **Figure 2**). Temperatures measured at 10 cm sediment depth were 75°C in the white and 26°C in the brown zone sediment. Temperature and pH measurements were not conducted in the transition zone sediment. The pore water concentration of hydrogen sulfide increased from 553 μM at a sediment depth of 2 cm to 608 μM at 4 cm; concomitantly, the sulfate concentration decreased from 11.3 to 10.5 mmol L^-^^1^ at the same depth.

The bulk seawater pH was 8.0 and the alkalinity 3.6 mmol kg^-^^1^. The pH in the white zone depth profile was between 5.2 and 5.3. Alkalinity decreased with depth from 2.1 to 1.1 mmol kg^-^^1^ in white sediment pore water (Price et al., 2012).

### SULFATE REDUCTION RATE MEASUREMENTS

**FIGURE 3:**
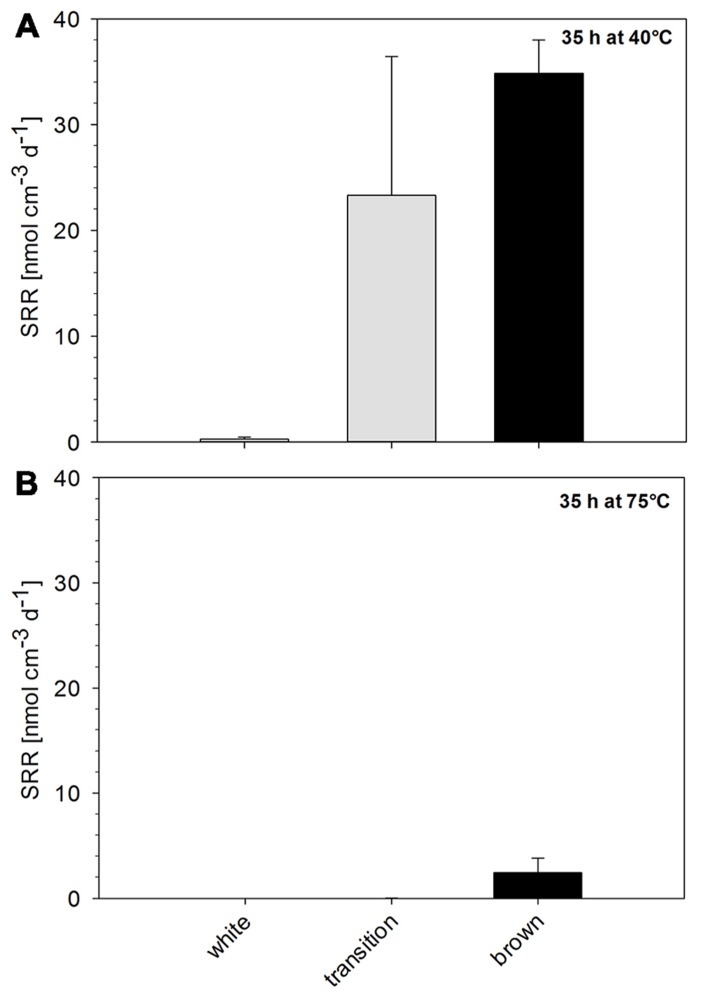
**Residual sulfate reduction rate of Paleochori Bay sediments after incubation for 35 h at a temperature of (A) 40°C and (B) 75°C**.

Sulfate reduction rates determined at 40°C in sediments that were collected close to the main venting site (white zone) were below 1 nmol ${SO}_{4}^{2-}$ cm^-^^3^ d^-^^1^. At this temperature, SRR increased with distance from the gas discharge zone, reaching 24 nmol ${SO}_{4}^{2-}$ cm^-^^3^ d^-^^1^ in surface sediment of the transition zone (**Figure 3A**). The highest activity at 40°C was found within the brown zone sediment (control) with 35 nmol ${SO}_{4}^{2-}$ cm^-^^3^ d^-^^1^. SR was mostly inhibited at 75°C (**Figure 3B**). However, SR was measurable in brown zone sediment at 75°C, with a SRR of 2.4 nmol ${SO}_{4}^{2-}$ cm^-^^3^ d^-^^1^, which is approximately 15 times less than the rate measured at 40°C. As SRR were below the detection limit of our method at 75°C, all further incubation experiments were conducted at an intermediate temperature of 40°C, which represents approximately the average found in hydrothermally influenced sediments.

### THE pH EFFECT ON SULFATE REDUCTION

**FIGURE 4:**
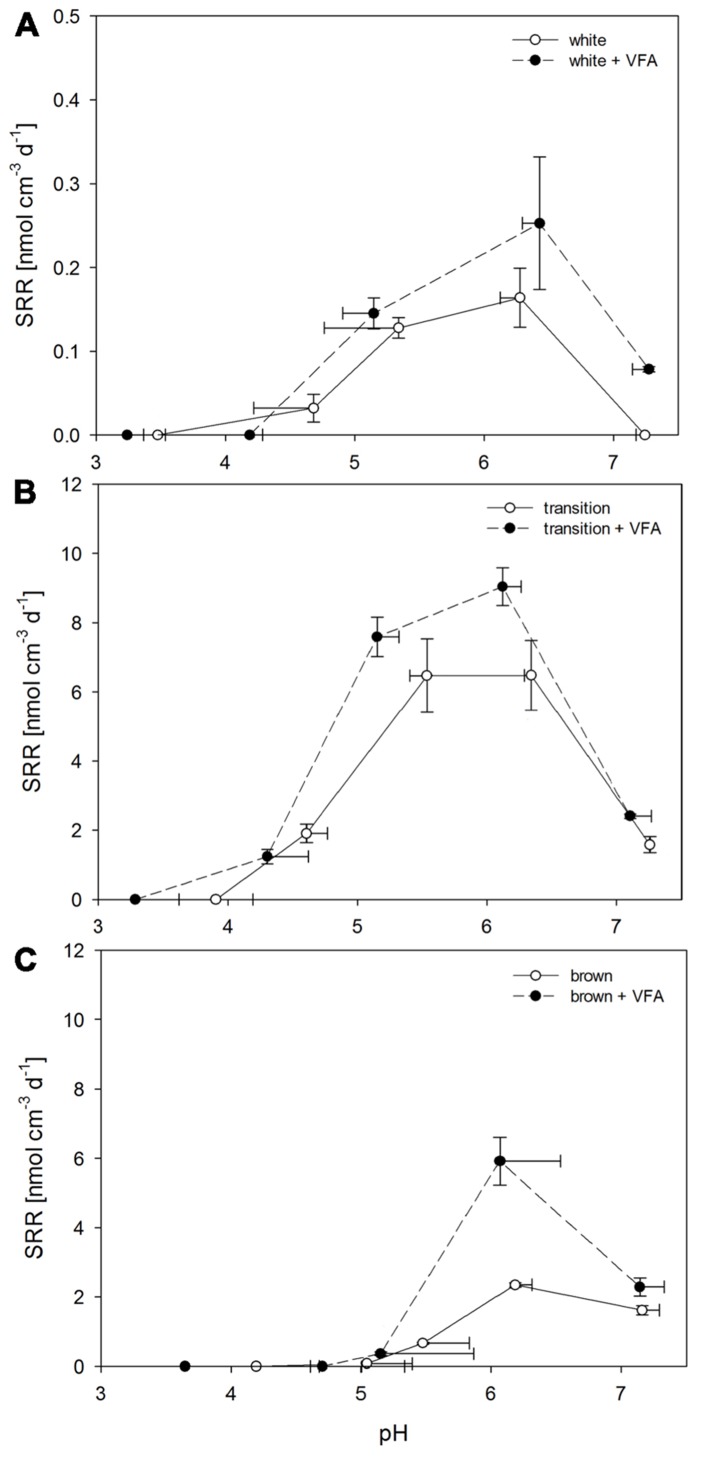
**Sulfate reduction rates after incubations for 16 h of Paleochori Bay, white, transition, and brown zone sediment incubated in not amended and VFA-supplemented media as a function of pH**. Incubation temperature was 40°C. Horizontal error bars show development of pH value over time of incubation. Vertical error bars show the standard errors of rates (*n* = 3). **(A)** White zone sediment. **(B)** Transition zone sediment. **(C)** Brown zone sediment.

The SRR increased with pH until they reached a pH optimum (pH_opt_) defined as the pH at which the highest SRR was measured (**Figures 4A–C**). The different sediment types of Paleochori Bay varied in the level of SR activity, its pH dependence and pH_opt_.

Among all tested sediments, the white zone sediment showed the lowest SR activity. The rates were statistically different for pH values between 3 and 7(*df* = 4, *F* = 17.47, *P* < 0.001). Highest rates were measured at pH 6.3 with an arithmetic average of 0.2 nmol ${SO}_{4}^{2-}$ cm^-^^3^ d^-^^1^ (**Figure 4A**). At pH 7 SRR were below the detection limit. This was also the case at pH < 4. At pH of 4.7 the rates were close to detection limit. The addition of 1 mmol L^-^^1^ of VFA to white zone sediment samples decreased SRR to below the detection limit at pH values < 4.5. The pH_opt_ remained at pH 6.4 but the corresponding SRR showed an increasing trend from 0.2 to 0.3 nmol ${SO}_{4}^{2-}$ cm^-^^3^ d^-^^1^ after VFA addition. SR activity could also be detected at pH 7.3 with a rate of 0.1 nmol ${SO}_{4}^{2-}$ cm^-^^3^ d^-^^1^ after VFA addition. In summary, the white zone sediment showed the highest SRR at pH values between 6.3 and 6.4 in treatments, both with and without VFA-amendment.

The transition zone sediments showed significantly different SRR for all pH values (*df* = 4, *F* = 19.89, *P* < 0.001; **Figure 4B**). The highest rate was measured between pH 5.5 and 6.3 with rates of 6.5 nmol ${SO}_{4}^{2-}$ cm^-^^3^ d^-^^1^. The SRR measured at pH_opt_ were 40 times higher in the transition zone as compared to the highest rates in white zone sediments. Furthermore, measurable rates at pH < 5 were found with 1.9 nmol ${SO}_{4}^{2-}$ cm^-^^3^ d^-^^1^ at a pH 4.6. At a pH < 4 the rates were below the detection limit. At pH 7.3 SRR were four times lower than at pH_opt_. After VFA-addition an increase in SRR could be identified as compared to the untreated slurries (*df* = 1, *F* = 5.02, *P* = 0.037) and the highest rates increased significantly from 6.5 nmol ${SO}_{4}^{2-}$ cm^-^^3^ d^-^^1^ (-VFA) to 9.0 nmol ${SO}_{4}^{2-}$ cm^-^^3^ d^-^^1^ (+VFA) at pH_opt_ of 6.1. The SRR at pH í 5.5 was slightly increased from 6.5 nmol ${SO}_{4}^{2-}$ cm^-^^3^ d^-^^1^ at a pH of 5.5 to a rate of 7.6 nmol ${SO}_{4}^{2-}$ cm^-^^3^ d^-^^1^ at pH 5.2 after amendment. At pH < 4, SR could no longer be detected. At pH > 7 the SRR were only increased from 1.6 nmol ${SO}_{4}^{2-}$ cm^-^^3^ d^-^^1^ without amendment to 2.4 nmol ${SO}_{4}^{2-}$ cm^-^^3^ d^-^^1^ after addition of VFA.

Sulfate reduction rates in the brown zone sediment were 2.3 nmol ${SO}_{4}^{2-}$ cm^-^^3^ d^-^^1^ at pH_opt_ of 6.0 (**Figure 4C**). Rates were significantly different between the tested pH values (*df* = 4, *F* = 213.73, *P* < 0.001). At pH 7.2 SRR showed a decrease from 2.4 to 1.6 nmol ${SO}_{4}^{2-}$ cm^-^^3^ d^-^^1^. In these sediment samples a steep decrease in SRR was observed at pH values < 6, reaching 0.7 nmol ${SO}_{4}^{2-}$ cm^-^^3^ d^-^^1^ at pH 5.5 and 0.1 nmol ${SO}_{4}^{2-}$ cm^-^^3^ d^-^^1^ at pH 5.0. At pH < 5, SRR were below the detection limit. VFA amendment resulted in a significant increase (*df* = 1, *F* = 26.06, *P* < 0.001) and a different response as compared to the other sediments. The pH_opt_ was 6.1 as was the case in the non-amended samples and the SRR increased from 2.3 nmol ${SO}_{4}^{2-}$ cm^-^^3^ d^-^^1^ with no amendment to 5.9 nmol ${SO}_{4}^{2-}$ cm^-^^3^ d^-^^1^ after VFA-addition. At pH 7 only a slight increase in SRR from 1.6 nmol ${SO}_{4}^{2-}$ cm^-^^3^ d^-^^1^ without amendment to 2.3 nmol ${SO}_{4}^{2-}$ cm^-^^3^ d^-^^1^ at after amendment was observed.

### THE pCO_2_ EFFECT ON SULFATE REDUCTION

**FIGURE 5:**
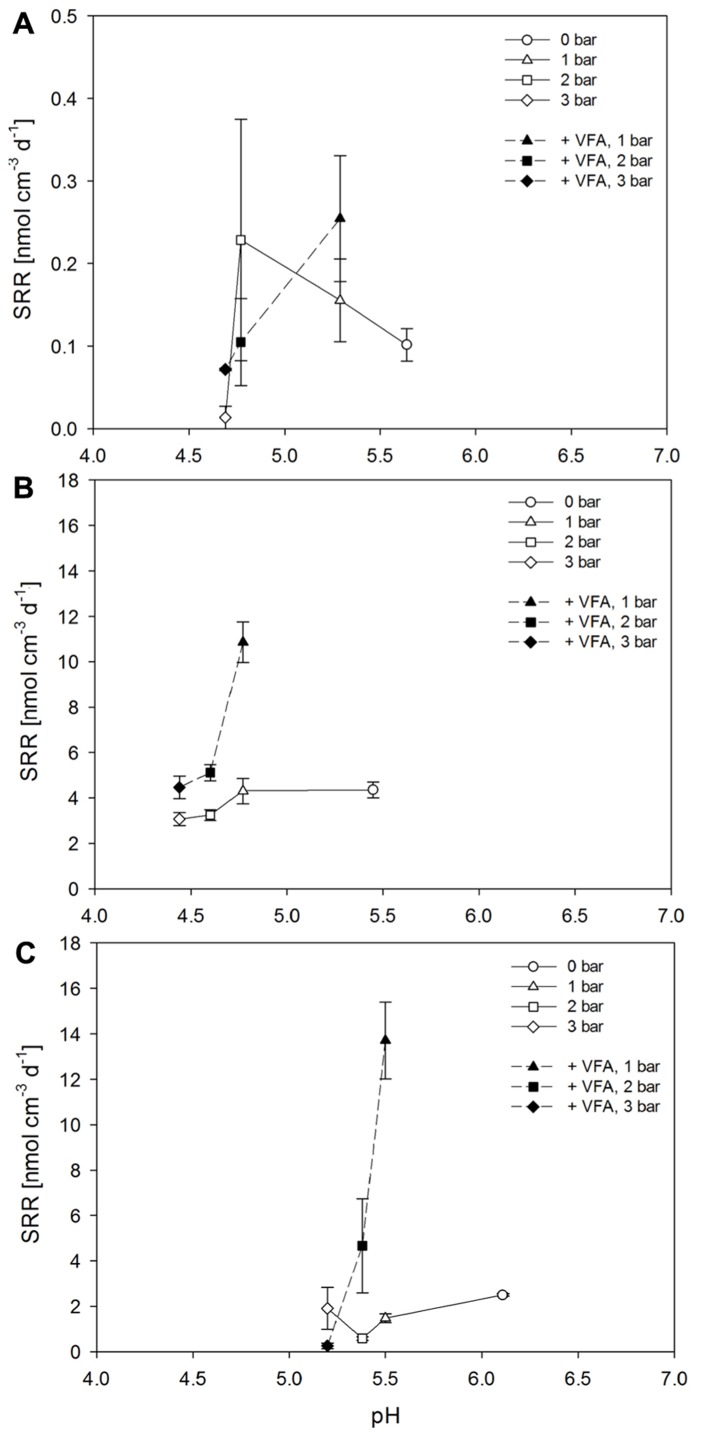
**Sulfate reduction rates after incubations for 16 h of Paleochori Bay, white (A), transition (B), and brown zone sediment (C) incubated in not amended and VFA-supplemented media as a function of changing pCO_2_**. Incubation temperature was 40°C. Vertical error bars show the standard errors of rates (n = 3). Symbols represent different pCO_2_ conditions, starting with circles for no pCO_2_, triangles for 1 bar, squares for 2 bar, and diamonds for 3 bar.

The different pCO_2_ partial pressures resulted in different pore water pH values in sediment samples obtained from the Paleochori Bay sites. In all sediment types a decreasing trend in SRR was observed with increasing pCO_2_ (**Figures 5A–C**). A stimulation of SRR upon VFA amendment was observed for all sediment types at a pCO_2_ of 1 bar. However, the rates decreased with increasing pCO_2_ after VFA addition and were significantly different for the transition (*df* = 2, *F* = 31.51, *P* < 0.001) and the brown zone sediment (*df* = 2, *F* = 19.68, *P* = 0.002). We observed a steep decrease of SRR in all samples after the CO_2_ gas pressure was increased from 2 to 3 bar even though the pH only decreased slightly, from 4.8 to 4.7 for the white zone sediment, from 4.7 to 4.6 for transition zone sediment and from 5.5 to 5.4 for the brown zone sediment (**Figures 5A–C**).

**FIGURE 6:**
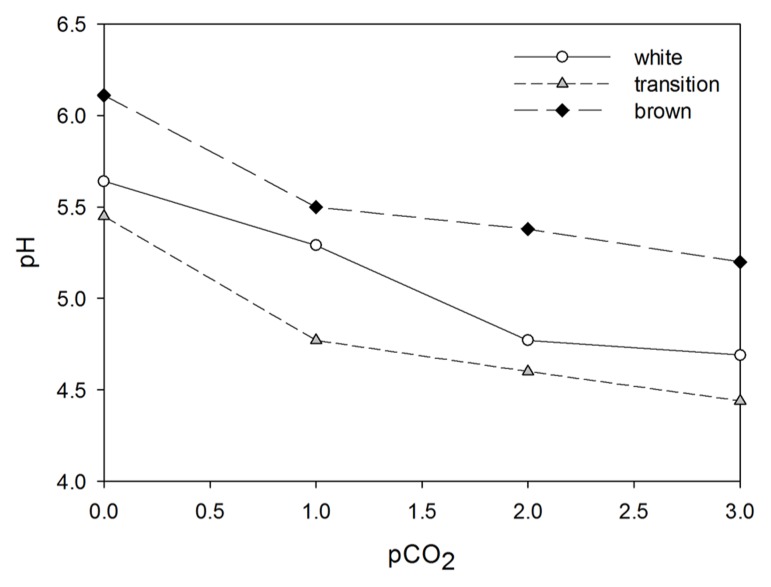
**Buffering capacity of white, transition, and brown sediment depicted by the pH of each sediment type to the corresponding pCO_2_** value.

The sediment samples showed different buffering capacities, which were expressed as the relation between pCO_2_ and subsequent pH decrease (**Figure 6**). The pore water of white zone sediment had an intermediate buffering capacity expressed by a pH decrease from 5.6 to 4.7 after addition of 3 bar of pCO_2_. The pore water of transition zone sediment had the lowest buffering capacity since the pH decreased from 5.5 to 4.4 after addition of 3 bar pCO_2_. The highest pH value and buffering capacity was found in samples from the brown zone sediment which decreased from 6.1 to 5.2 after pCO_2_ adjustment (**Figure 6**).

## DISCUSSION

In this study, the highest SRR without VFA amendment were measured in the brown zone sediment with 35 nmol ${SO}_{4}^{2-}$ cm^-^^3^ d^-^^1^. SRR determined in these control sediment samples were close to rates reported from temperate regions. For example, Jørgensen and Bak (1991) reported SRR between 5 and 20 nmol ${SO}_{4}^{2-}$ cm^-^^3^ d^-^^1^ from marine sediments of Kattegat, Denmark. Dando et al. (1995) studied a neighboring hydrothermal vent site in Paleochori Bay at a water depth of 10 m and determined SRR of between 5 and 80 nmol ${SO}_{4}^{2-}$ cm^-^^3^ d^-^^1^ applying a radiotracer method (Dando et al., 1991) immediately after sampling. SRR peaked at 2 cm sediment depth with maximal rates of up to of 80 nmol ${SO}_{4}^{2-}$ cm^-^^3^ d^-^^1^, which were probably supported by residual organic carbon (Dando et al., 1995) and/or hydrogen gas from the vent fluids. The overall lowered rates in our sediment samples were most likely a consequence of electron donor limitation as the experiments could not be performed immediately after sampling. Intermediate activity was found in surface samples of transition zone sediment with a SRR of 24 nmol ${SO}_{4}^{2-}$ cm^-^^3^ d^-^^1^. SRR in brown and transition zone sediment were in the range of SR determined in deep-sea hydrothermal vent sediments (19–61 nmol ${SO}_{4}^{2-}$ cm^-^^3^ d^-^^1^) with *in situ* temperatures > 100°C (Jørgensen et al., 1992) but below the rates measured for the Logatchev hydrothermal field at the Mid Atlantic Ridge with 122–136 nmol ${SO}_{4}^{2-}$ cm^-^^3^ d^-^^1^ at temperatures between 65 and 100°C (Schauer et al., 2011). The lowest SRR was measured in the white zone sediment. There, SRR were below 1 nmol ${SO}_{4}^{2-}$ cm^-^^3^ d^-^^1^ after incubation at 40°C and below detection limit when the samples were incubated at 75°C, which is the *in situ* temperature at 10 cm sediment depth. The low SRR in the white zone may be due to substrate limitation, in particular lack of hydrogen gas from venting fluids.

The SRB are found in several phylogenetic groups, but mostly belong to the Deltaproteobacteria (e.g., 23 out of 60 genera), followed by the Firmicutes (Muyzer and Stams, 2008; Barton and Fauque, 2009). It is possible that the low SRR, especially in the white zone sediments, may be due to a smaller population size of SRB, at least relative to our other sediment types. Sievert et al. (1999) found mainly Cyanobacteria and sulfur oxidizing *Thiomicrospira* spp in a hydrothermal vent system that is located close to our study site. Our sediments were collected from a slightly higher temperature, 75°C at 10 cm depth, whereas the white zone temperatures for Sievert et al. (1999) study reached only 50°C at 5 cm sediment depth. Even if polymerase chain reaction (PCR) biases cannot be excluded, the elevated numbers of 16S rRNA sequences affiliated with the Deltaproteobacteria, to which most SRB belong, found in the transition and brown zone sediments (Sievert et al., 1999) are in agreement with our results as we found the highest SRR in these sediments.

In a more quantitative study, 16S rRNA clone libraries and phylogenetic data were obtained for samples collected in the same area and temperature as this study (Price et al., 2013, this issue). In the white zone with equivalent temperatures to our study, 7 out of 51 clones were obtained for the Deltaproteobacteria in a 0–1.5 cm sediment depth, whereas none were found in the deeper 3–4.5 and 9–10.5 cm layers. Four out of those seven clones were affiliated with the order Desulfobacterales, one of the major groups known for SR (Muyzer and Stams, 2008). Price et al. (2013, this issue) also reported that members of the Firmicutes phylum became dominant in the deeper sediments, but were exclusively associated with *Bacillus* sp (at ~100% ID). In a pooled 0–6 cm sample from a lower temperature (~45°C) white zone at the same site as this study, 24 out of 119 clones were affiliated with Deltaproteobacteria, 12 of which were associated with the Desulfobacterales order. In pooled 0–6 cm sample from a nearby background brown area, 10 out of 97 clones were affiliated with Desulfobacterales. Thus, in pooled samples of brown zone sediment, about 10% of the clones in the clone libraries affiliate with groups with the most potential for ${SO}_{4}^{2-}$ reduction, while 10% affiliated with SRBs in white zone sediments. In the 0–1.5 cm sediment sample, the contribution of 16S rDNA affiliated with SRB was 8%. Due to the small size of the libraries we do not consider the ratios to significantly different among sites. In future studies the microbial diversity in general and that of sulfate reducers should be addressed with next generation sequencing methods (Frank et al., 2013).

A possible explanation for the low SRR in white zone sediment could also be its high spatial instability due to transport of gas, venting and mixing in combination with oxygenation of the sediment layers which could lead to a removal of microorganisms. Mixing might also disturb the coupling between fermenters and sulfate reducers, which thrive on oxidizing fermentation products such as VFA and H_2_. Finke and Jørgensen (2008) observed that temperature had an effect on the coupling between fermentation and SR in sediment samples from Svalbard and Wadden Sea as fermentation products accumulated in incubations above a critical temperature of 40°C. The authors concluded that temperature is a factor that might disturb the metabolic link between both process types because sulfate reducers were inhibited at a lower critical temperature than fermenters (Finke and Jørgensen, 2008). In our study, a higher threshold for negative temperature effects is expected as the natural temperatures of the hydrothermal vent system ranged from 26 to 75°C. Effects that reduce SRR can most likely be attributed to low pH leading to a protonation of acid anions, which short-cuts proton gradients in the cells and disrupts adenosine triphosphate (ATP) synthesis (Baronofsky et al., 1984).

### THE pH EFFECT ON SULFATE REDUCTION

Based on cell culture studies, it was suggested that SR may preferentially occur at pH between 6 and 8 (Widdel, 1988; Hao et al., 1996). However, Dando et al. (1995) have documented that SR can take place at high rates in shallow submarine hydrothermal vent systems with low pH. Our results are in line with those reported by Dando et al. (1995), and demonstrate that hydrothermal sediments with lower pH and increased temperature harbor populations of SRB that respond distinctly to different pH conditions. In addition, our data indicate that hydrothermal sediments had different pH optima than SR populations of sediments with low temperature and neutral to slightly alkaline pH characteristics. We observed SR at pH < 5 in hydrothermally influenced, CO_2_- vented sediments. However, optimal pH of SR was found between pH 5 and 6 for the hydrothermally influenced white and transition zone sediments but between pH 6–7 for brown zone sediment that served as control.

Sulfate reducing activity under low pH conditions is often explained by the existence of microenvironments with more reduced and alkaline conditions than the acidic surroundings (Fortin, 1996). Koschorreck (2008) argued against the existence of neutral microniches formed by the alkalinity produced during SR. He calculated that a SRR of 1.8 × 10^8^ nmol ${SO}_{4}^{2-}$ cm^-^^3^ d^-^^1^ is required to maintain circumneutral pH in a sphere with 100 μm diameter while the pH in the surrounding is 3. Applying this calculation to the hydrothermal vent sediments of Paleochori Bay, a SRR of 1.6 × 10^6^ nmol ${SO}_{4}^{2-}$ cm^-^^3^ d^-^^1^ would be needed to maintain the center of the sphere at pH 6 when the pH of the surrounding pore water was 5. This rate exceeds the SRR in the hydrothermal vent system of Paleochori Bay by five to six orders of magnitude and supports our argument that SRB populations are adapted to low pH rather than the presence of neutral microsites.

In previous studies, it was suggested that sulfate reducers are more susceptible to high VFA concentrations than methanogens (James et al., 1998), which might result in a shift from SR to methanogenesis at low pH (Koschorreck, 2008). A study on methanogenesis in a bioreactor at pH of 4.5 showed a 30% increase of methane yield as compared to neutral conditions, after a slow acclimation of the methanogens to lowered pH (Taconi et al., 2008). A competition between sulfate reducers and methanogens due to diluted sulfate concentrations by the vent outflow and higher susceptibility of the sulfate reducing community to low pH, can be excluded as the abundance of Archaea as well as their diversity was low in white and brown zone sediments (Nitzsche, 2010; Price et al., 2013, this issue).

### THE EFFECT OF VOLATILE FATTY ACIDS ON SULFATE REDUCTION AT LOW pH

Protonated short-chained fatty acids diffuse through the cell membrane and consequently act as protonophores and as uncoupling agents (Kell et al., 1981). This is not the case for their conjugate anions, which are excluded by their physical properties and charged head groups of lipids from the biological bilayer. Small fatty acids turn lipophilic under acidic conditions depending on their dissociation constant pK*a*, pass through membranes by passive diffusion and destruct the gradients (pH gradient, ΔpH and membrane potential ΔΨ) necessary for ATP synthesis and transport function. In addition, they acidify the intrinsic circumneutral cytoplasm of the cell by dissociation and proton release. The reduced cytoplasmic pH inhibits cellular reactions and energy conservation triggered by proton-motive force (Baronofsky et al., 1984). With the formula:

$$[Ac]/[HAc]\;=\;10^{pH\,-pKa}$$

where [Ac] equals the total acid concentration and [HAc] the amount of protonated acid at a certain pH and the pK*a* values of a total concentration of 1 mmol L^-^^1^ VFA (formate, acetate, propionate, butyrate, and succinate) with pK*a* values of 3.75; 4.76; 4.87; 4.81; 4.16 (Perrin, 1972) in the slurry we can calculate the concentration of protonated fatty acids (Bruun et al., 2010) at pH values from 3 to 7. The total concentration of protonated organic acids of the VFA in the slurry is 43.3 mmol L^-^^1^ at pH of 3; 4.3 mmol L^-^^1^ at pH 4, 0.4 mmol L^-^^1^ at pH 5; 0.01 mmol L^-^^1^ at pH 6 and negligible for a pH 7 (0.004 mmol L^-^^1^). In this study, only the protonated fatty acid concentration at pH values of 3 and 4 might become harmful to SRB (Baronofsky et al., 1984). This is consistent with our pH-experiment data in which the SRR of the VFA-amended slurry is lower than the rate measured in unamended sediment of white and transition zone sediment at pH < 5. At pH 6.4, the SRR measured after VFA addition to white zone sediment (0.3 nmol ${SO}_{4}^{2-}$ cm^-^^3^ d^-^^1^) exceeded the SRR (0.2 nmol ${SO}_{4}^{2-}$ cm^-^^3^ d^-^^1^) at a similar pH (pH 6.3) without addition. In brown zone sediment slurries, the limit for VFA stimulation was equal to unamended sediment at pH 5.5. The SRR at pH 6.2 of 2.3 nmol ${SO}_{4}^{2-}$ cm^-^^3^ d^-^^1^ was stimulated by VFA amendment to a rate of 5.9 nmol ${SO}_{4}^{2-}$ cm^-^^3^ d^-^^1^. A SRR increase was also observed at neutral pH for brown zone sediments: here SRR increased from 1.6 nmol ${SO}_{4}^{2-}$ cm^-^^3^ d^-^^1^ without amendment to 2.3 nmol ${SO}_{4}^{2-}$ cm^-^^3^ d^-^^1^ in amended slurries. Since VFA were added as a mixture to the slurries, no further differentiation between the individual fatty acids on SRR was possible. The presented results are consistent with studies on activity inhibition caused by organic acids at artificially lowered pH (Ottosen et al., 2009) and fresh water seep systems (Bruun et al., 2010).

### THE EFFECT OF pCO_2_ ON SULFATE REDUCTION

The content of CO_2_ correlates with pH. Dissolved CO_2_ reacts with water to form carbonic acid (H_2_CO_3_), which immediately dissociates to bicarbonate (HCO_3_^-^) and protons (H^+^). HCO_3_^-^ and H^+^ can further dissociate to ${CO}_{3}^{2-}$ and H^+^ decreasing the pH in the solution. In this context, “carbonic acid” encompasses the species CO_2_, H_2_CO_3_, HCO_3_^-^, and ${CO}_{3}^{2-}$ (Zeebe and Wolf-Gladrow, 2001). The existence of the different carbonic acid species depends on their concentrations, dissociation constants and pH as described by the Bjerrum plot (Zeebe and Wolf-Gladrow, 2001).

In this study, the relation between pH and SRR relative to different pCO_2_ was examined. This is of particular interest in order to understand microbial functioning in CO_2_ – venting sediments which are perfect natural laboratories for studies focused on CO_2_ sequestration in which carbon dioxide is removed from the atmosphere and stored in sediments that should serve as long-term reservoirs (Shitashima et al., 2008). After adjustment of the CO_2_ partial pressure to 0, 1, 2, and 3 bar, we found that a pCO_2_ increase resulted in different pH values among the sediment types. This may be a consequence of different chemical and biological buffering capacities of the sediment (Heijs et al., 1999). The mineral characteristics of the sediment (e.g., silicate, carbonate) and the bicarbonate content of the seawater dictate chemical buffering capacity of the sediment. The addition of CO_2_ results in a decrease in pH (Zeebe and Wolf-Gladrow, 2001). SR produces bicarbonate alkalinity and consequently counteracts a decrease of pH. The data obtained from pCO_2_ incubation experiments provide a rough indication of the buffering capacity of the Paleochori Bay sediments: the brown zone sediment has the highest buffering capacity followed by white and finally transition zone sediments. This was also obvious from the amounts of phosphoric acid needed to decrease the pH in the pH experiments (data not shown).

Sulfate reduction rates responded to increase in pCO_2_ with a decrease in activity in nearly all experiments although effect of an increase in pCO_2_ from 2 to 3 bar on pH was negligible. A pCO_2_ increase from 1 to 2 bar reduced SRR from 0.2 to 0.1 nmol ${SO}_{4}^{2-}$ cm^-^^3^ d^-^^1^ in the white zone sediment slurries, from 4.3 to 3.2 nmol ${SO}_{4}^{2-}$ cm^-^^3^ d^-^^1^ in transition zone slurries and from 1.5 to 0.6 nmol ${SO}_{4}^{2-}$ cm^-^^3^ d^-^^1^ for the brown zone sediment slurries. The increase in pCO_2 _was accompanied by pH changes from 4.8 to 4.7, 4.8 to 4.6 and 5.5 to 5.4 in the three sediments, respectively. The effect of SRR reduction upon pCO_2_ increase was even stronger after VFA were added to the slurries (**Figure 5**). In our experiments we observed similar effects as in Debs-Louka et al. (1999) concluding that the reduction in pH due to increasing pCO_2_ was not sufficient to account for antimicrobial activity. In this study microbial inactivation depended strongly on CO_2_ partial pressure (tested for 15–55 bar), exposure time, the decompression time and water content of the sample. A sudden increase in pCO_2_ was observed to provoke cell ruptures and lead to reduced bacterial numbers (Debs-Louka et al., 1999). Additionally, it can be suggested that the content of CO_2_ in the form of carbonic acid has a negative effect on the cell as it can permeate the membrane and dissociate in ${CO}_{3}^{2-}$ and H^+^ in the circumneutral cytoplasm, decreasing the intrinsic pH of the cell.

## CONCLUSION

We observed a significant difference between SR in hydrothermally influenced and background sediments suggesting that microbial communities are adapted to low pH in the hydrothermal sediments of Milos. Sulfate reducing communities in hydrothermal sediments showed pH activity optima between 5 and 6. In contrast, SR in sediments with no hydrothermal influence exhibited pH optima between 6 and 7. The shallow-sea hydrothermal vent of Paleochori Bay, Milos (Greece) with pH between 5 and 7, temperature range of 26–90°C and a high content of dissolved arsenic and sulfide in the sediment pore water, represents an extreme environment in which SR may play an important role in the degradation of organic material. This study suggests that marine microbial SR communities that are specifically adapted to a life at high pCO_2_ and low pH do exist.

## Conflict of Interest Statement

The authors declare that the research was conducted in the absence of any commercial or financial relationships that could be construed as a potential conflict of interest.
